# Advances in Cellular Characterization of the Sirtuin Isoform, SIRT7

**DOI:** 10.3389/fendo.2018.00652

**Published:** 2018-11-19

**Authors:** Di Wu, Yinglu Li, Kathy S. Zhu, Haiying Wang, Wei-Guo Zhu

**Affiliations:** ^1^Key Laboratory of Carcinogenesis and Translational Research (Ministry of Education), Beijing Key Laboratory of Protein Posttranslational Modifications and Cell Function, Peking University Health Science Center, School of Basic Medical Sciences, Beijing, China; ^2^School of Basic Medical Sciences, Institute of Systems Biomedicine, Peking University Health Science Center, Beijing, China; ^3^Department of Biochemistry and Molecular Biology, Shenzhen University Health Science Center, Shenzhen, China; ^4^Peking University Health Science Center, School of Public Health, Beijing, China; ^5^Department of Biochemistry and Molecular Biology, Peking University Health Science Center, School of Basic Medical Sciences, Beijing, China

**Keywords:** SIRT7, rDNA transcription, ribosome biogenesis, cellular stress, metabolism, aging, genome stability, cancer

## Abstract

SIRT7 is one of seven mammalian sirtuins that functions as an NAD^+^-dependent histone/protein deacetylase. SIRT7 is the least well-known member of the sirtuin family, but recent efforts have identified its involvement in various cellular processes, such as ribosome biogenesis, gene expression, cellular metabolism and cancer. Here we provide an update on the functions and mechanisms of SIRT7 in cellular regulation and disease.

## Introduction

**Figure 1:**
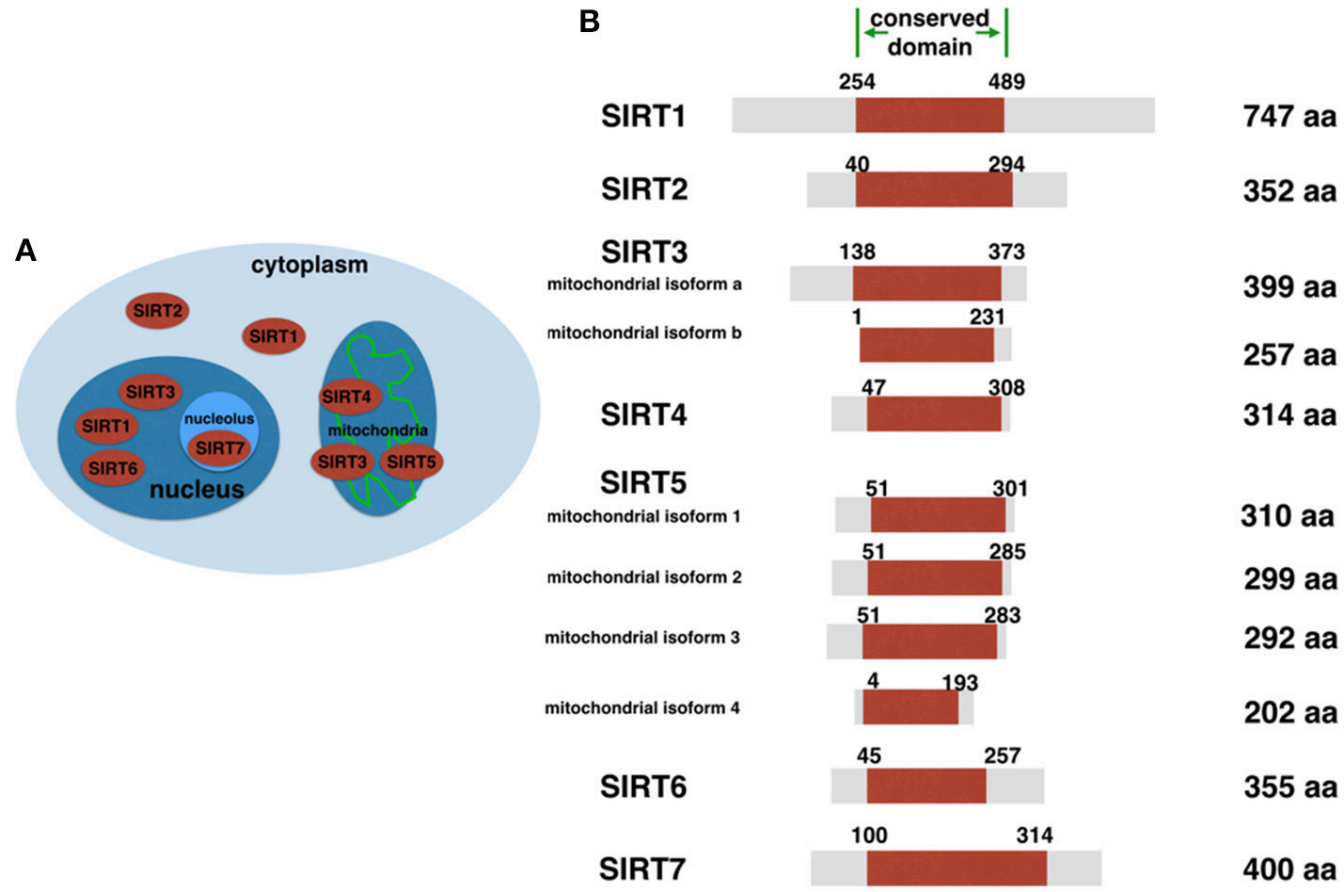
Sirtuin family. **(A)** Sirtuins' intracellular location. **(B)** Human sirtuin proteins and their protein structures. The sequences are based on NCBI Protein database.

In the year 2000, researchers identified that silent information regulator 2 (Sir2)—a nicotinamide adenine dinucleotide (NAD)-dependent histone deacetylase—extends the lifespan of yeast (1, 2). Since then, the Sir2 homologs in mammals, known as sirtuins, have received increasing attention. There are seven sirtuin homologs in mammals (SIRT1 to SIRT7) that share a conserved NAD^+^ binding domain, but their cellular localization, activities and functions differ (Figure 1). SIRT1, SIRT6, and SIRT7 predominantly localize to the nucleus (SIRT7 primarily resides in nucleolus), SIRT2 localizes to the cytoplasm and SIRT3, SIRT4, and SIRT5 localize to the mitochondria (3). Sirtuins mediate various cellular functions that regulate a wide range of physiological processes, including the cell cycle, proliferation, apoptosis, aging, genomic stability, stress resistance and metabolism (4). SIRT7 is the least well-studied of the sirtuins, but recent breakthroughs have shown that it is also involved in numerous cellular processes and its biological function is gradually becoming clear. In this review, we outline the current data regarding SIRT7 function and highlight the areas where SIRT7 may have a possible therapeutic role in disease.

## SIRT7 enzymatic activity

*SIRT7* encodes a 400 amino acid protein that functions as a class III histone deacetylase in *Homo sapiens*. (5) Compared to other nuclear-localized Sirtuins (SIRT1 and SIRT6), SIRT7 exhibits weak deacetylase activity (3). Within the SIRT7 catalytic domain, serine residue 111 (S111) and histidine residue 187 (H187) are responsible for the deacetylation activity (6, 7), but to date, only a few SIRT7 substrates have been identified. In 2012, Barber et al. (6) reported that SIRT7 is a highly selective histone H3 lysine 18 (H3K18) deacetylase, and that H3K18Ac deacetylation is associated with high-grade tumors and poor patient prognosis. Non-histone substrates have also been identified, including p53, PAF53, U3-55k, GABPβ1, NPM1, PGK1, CDK9, DDB1, FKBP51, FOXO3, SMAD4, and DDX21 (Table 1). We discuss the data for each of these substrates in turn below.

**Table 1:** SIRT7 catalytic substrates.

**Substrate**	**Modification**	**Function**
H3K18	Deacetylation	Tumorigenesis
p53	Deacetylation	Apoptosis
PAF53 and U3-55k	Deacetylation	Synthesis and ripening of precursor ribose RNA
GABPβ1	Deacetylation	Mitochondrial function
NPM1	Deacetylation	Aging
PGK1	Deacetylation	Glycolysis
FKBP51	Deacetylation	AKT inactivation and chemo-sensitivity in breast cancer cells
CDK9	Deacetylation	Transcription elongation
FOXO3	Deacetylation	Blocks apoptosis in response to LPS
DDB1	Deacetylation	Regulates DDB1-CUL4 interaction and CRL4 activity
SMAD4	Deacetylation	Inhibits breast cancer lung metastasis
DDX21	Deacetylation	Genome stability
H3K122	Desuccinylation	Chromatin remodeling during DNA repair
H3K36/K37	Deacetylation/debutyrylation	Unknown

Vakhrusheva et al. found that p53 peptides were deacetylated *in vitro* by SIRT7 as efficiently as SIRT1, and mutant SIRT7 nearly abolished p53 decetylation (8). Conversely, Barber et al. did not detect SIRT7 deacetylase activity toward p53 both *in vivo* and *in vitro*, which questioned the ability of SIRT7 to deacetylate p53 (6). Kim et al. found an inverse correlation between p53 acetylation levels and SIRT7 expression in Hep3B cells (9). Finally, Nahalkova et al. identified an interaction between SIRT7 and p53, but did not assess SIRT7 deacetylase activity (10). p53 is debatable to be a SIRT7 substrate, for SIRT7 is found only effective toward p53 peptide, but not p53 protein both *in vivo* and *in vitro*. More studies are required, therefore, to resolve whether p53 is truly a SIRT7 substrate.

The wide variety of non-histone SIRT7 substrates suggests that SIRT7 participates in a diverse range of cellular processes. For example, SIRT7 can deacetylate nucleolar organizer polymerase-associated factor 53 (PAF53) and U3-specific protein U3-55k to regulate the synthesis and ripening of precursor ribose RNA (11, 12). By bioinformatic analysis, Ryu et al. found that GA-binding protein β1 (GABPβ1) is another substrate of SIRT7 (13). Here, SIRT7 interacts with and deacetylates GABPβ1 to regulate mitochondrion function (13). Lee et al. reported that SIRT7 deacetylates nucleophosmin (NPM1), which is involved in regulating aging processes (14), while Hu et al. found that SIRT7 deacetylates phosphoglycerate kinase 1 (PGK1) and participates in glycolysis regulation (15). Yu et al. showed that SIRT7 specifically interacts with and deacetylates FK506-binding protein 51 (FKBP51) at lysine residues 28 and 155 (K28 and K155), which enhances FKBP51–Akt–PHLPP complex formation, resulting in AKT inactivation and enhanced chemo-sensitivity in breast cancer cells (16). Blank et al. showed that SIRT7-dependent deacetylation of Cyclin-dependent kinase 9 (CDK9) activates its kinase activity; CDK9 subsequently phosphorylates the Pol II C-terminal domain (CTD) and facilitates transcription elongation (17). Li et al. observed that SIRT1/SIRT7 deacetylate FOXO3 *in vitro* and *in vivo* to prevent its phosphorylation and block apoptosis in response to lipopolysaccharide stimulation (18). Mo et al. found that SIRT7 is a major deacetylase of DNA damage-binding protein 1 (DDB1): DDB1 acetylation negatively regulates the DDB1–CUL4 interaction and CRL4 activity (19, 20). Tang et al. found that SIRT7 inhibits breast cancer lung metastasis by deacetylating and promoting SMAD4 degradation (21). Finally, Song et al. showed that deacetylation of the nucleolar DEAD-box helicase DDX21 by SIRT7 increases R-loop-unwinding activity and safeguards genome stability (22).

To widen the range of SIRT7 targets, our laboratory conducted the first proteomic screen of SIRT7 substrates. Using stable isotope labeling with amino acids in cell culture coupled with quantitative mass spectrometry, we produced a comprehensive list of candidates that are involved in a variety of functions, ranging from chromatin architecture homeostasis to gene silencing and metabolism. Some selected candidates, such as SIRT2, histone-lysine N-methyltransferase (EZH2), mitogen-activated protein kinase 2 (MAPK2) and glycogen synthase kinase-3 beta (GSK3β) have been validated by *in vitro* co-immunoprecipitation and deacetylation experiments. By combining this approach with predictive tools, we have started to greatly expand the list of SIRT7 candidate substrates. Such studies have enhanced our understanding of the physiological role of SIRT7 and raised awareness as to the global impact of sirtuins in cell homeostasis (23).

SIRT7 exhibits other enzymatic activities in addition to NAD^+^-dependent deacetylation. In 2016, Li et al. found that SIRT7 is an NAD^+^-dependent histone desuccinylase, and desuccinylates H3K122 to regulate chromatin remodeling during DNA repair (24). However, no non-histone substrates for SIRT7 desuccinylase have been identified. SIRT7 also interacts with some proteins without deacetylating them, such as mTOR, Myc, and TFIIIC2 (25); it is possible that SIRT7 regulates these proteins via its desuccinylase activity. Future investigations should investigate the spectrum of non-histone desuccinylation-mediated regulation by SIRT7. A recent report also showed that SIRT7 has robust deacetylase or debutyrylase activity on acetylated or butyrylated nucleosomes, mainly toward H3K36/K37 (26).

SIRT7 deacetylase activity is markedly enhanced by chromatin compositional DNA and RNA. SIRT7 also exhibits defatty-acylase activity, which can be effectively activated by RNA (27, 28). Conversely, Myb-binding protein 1a (Mybbp1a), a component of the chromatin remodeling complex B-WICH, inhibits SIRT7 deacetylation activity and increases H3K18 levels in cells (29), although the mechanistic details remain to be understood.

## SIRT7 expression and regulation

SIRT7 is widely expressed in different organs and tissues of the human body: the highest expression is found in hyperplastic tissues, such as the spleen, liver and testis, and the lowest expression is found in the skeletal muscle, heart and brain (30). SIRT7 expression levels are associated with cellular proliferation, differentiation and the stress response, acting as a positive or negative regulator in different organs and tissues (31). Recent findings from the TCGA database suggest that SIRT7 expression is tightly correlated with the development of various types of cancer (32–34). The diverse roles of SIRT7 suggest it is tightly regulated by multiple mechanisms, as evidenced by SIRT1 (35, 36).

**Figure 2:**
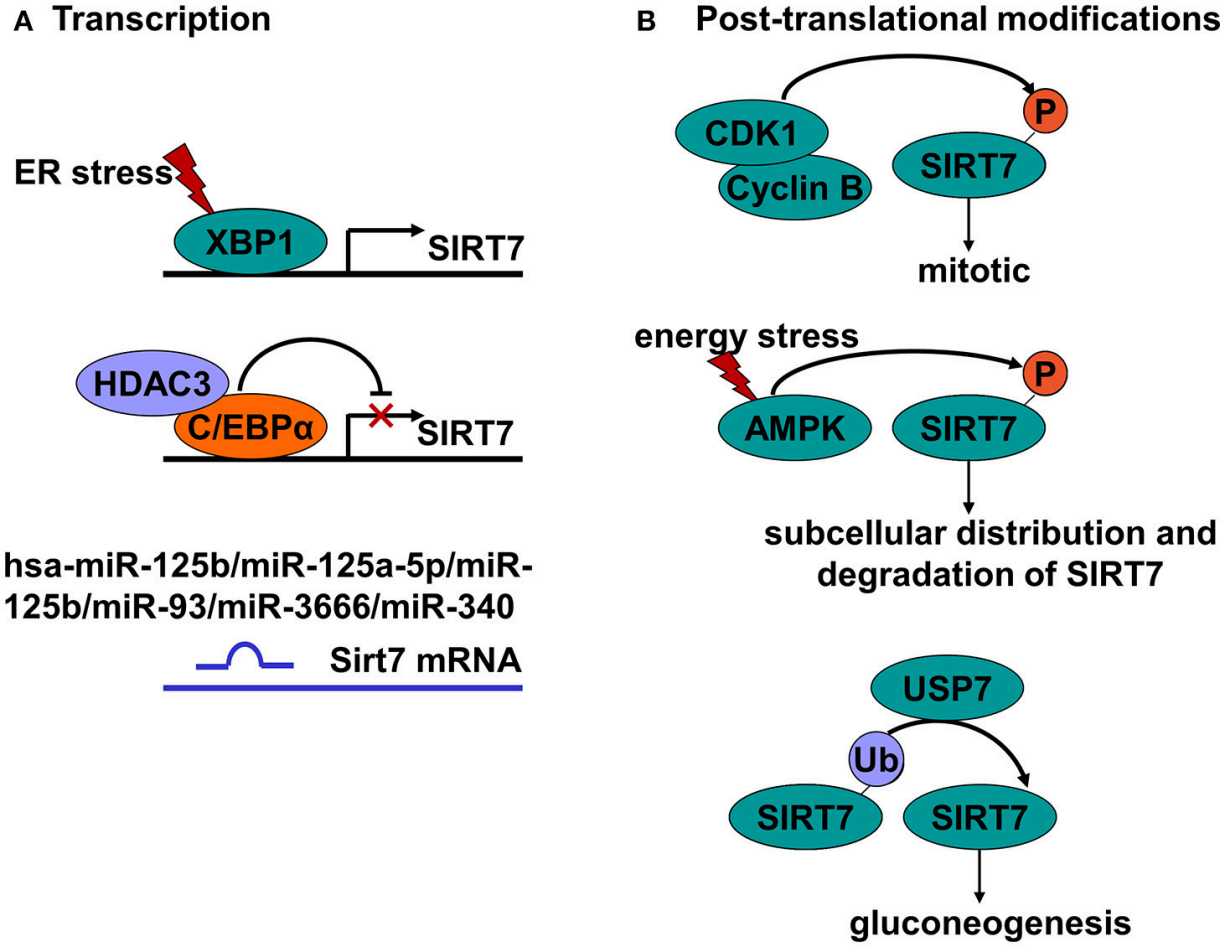
Summary of regulation of SIRT7. **(A)** Various transcription factors regulate SIRT7 expression. The X-box binding protein 1 (XBP1) enhances SIRT7 expression, whereas Histone Deacetylase 3 (HDAC3) and CCAAT/enhancer-binding protein alpha (C/EBP alpha) repress SIRT7 expression. In addition, SIRT7 expression is also repressed by the microRNAs, such as hsa-miR-125b, miR-125a-5p, miR-125b, miR-93, miR-3666, and miR-340. **(B)** Some post-translational modifications affect SIRT7 activity. The cyclin-dependent kinase 1 (CDK1)–cyclin B pathway phosphorylates SIRT7 during mitosis. Under energy stress, SIRT7 can be phosphorylated by Adenosine 5′-monophosphate (AMP)-activated protein kinase (AMPK), thereby determining the subcellular distribution and degradation of SIRT7. Moreover, Ubiquitin-specific protease 7 (USP7) negatively regulates the enzymatic activity of SIRT7 through deubiquitination to control gluconeogenesis.

Previous reports have indicated that SIRT7 is regulated at the transcriptional, post-transcriptional and translational levels (Figure 2). At the transcriptional level, SIRT7 is regulated by upstream molecules, such as X-box binding protein 1 (XBP1), CCAAT-enhancer-binding protein α (C/EBPα), and histone deacetylase 3 (HDAC3) (37, 38). At the post-transcriptional level, SIRT7 is negatively regulated by several microRNAs, such as hsa-miR-125b, miR-125a-5p, miR-125b, miR-93, miR-3666, and miR-340 (9, 39–42). There are only few reports, however, describing how SIRT7 is regulated by post-translational modifications. Grob et al. found that SIRT7 is phosphorylated during mitosis by the cyclin-dependent kinase 1 (CDK1)-cyclin B pathway, although the phosphorylation sites and function have not been defined (43). Sun et al. found that SIRT7 is phosphorylated during cellular energy stress by 5′ AMP-activated protein kinase (AMPK), which has a crucial role in determining SIRT7 subcellular distribution and degradation (44). The researchers also showed that SIRT7 is modified by polyubiquitination (44), which is consistent with our study that showed that SIRT7 is modified by Lys-63-linked polyubiquitination (45). We also found that SIRT7 enzymatic activity is negatively regulated by ubiquitin-specific-processing protease 7 (USP7)-mediated deubiquitination, which helps control gluconeogenesis by regulating *G6PC* expression (45). Post-translational modifications have a crucial role in regulating protein stability, activity, structure and function; thus, further studies into SIRT7 post-translational modifications are warranted.

## SIRT7 biological functions

### Ribosome biogenesis and protein translation

Global proteomic studies have identified numerous SIRT7-associated proteins, with most of them having important roles in transcription, ribosome biogenesis and translation (7, 14, 25). Subsequent functional studies have confirmed that SIRT7 is involved in multiple pathways regulating ribosome biogenesis and protein translation. First, SIRT7 can regulate rDNA transcription. Ford et al. found that SIRT7 forms part of the RNA Polymerase I (RNA Pol I) complex, and activation of the RNA Pol I transcription *in vivo* is dependent on its enzymatic activity (30). Specifically, the researchers showed that SIRT7 over-expression increased Pol I-mediated transcription, whereas SIRT7 knockdown or catalytic inhibition decreased Pol I transcription (30). Grob et al. also found that SIRT7 activates Pol I transcription by interacting with upstream binding factor (UBF), which is a component of the RNA polymerase complex (43). Tsai et al. extended the mechanisms of SIRT7-mediated rDNA transcriptional regulation by showing that SIRT7 associates with the B-WICH complex, a chromatin remodeling complex involved in rDNA transcriptional regulation and chromatin structure changes. In addition, the researchers found that down-regulated SIRT7 reduced levels of RNA Pol I protein by decreasing the expression levels of RPA194, the largest RNA polymerase I subunit (7). Others have shown that SIRT7 regulates Pol I transcription by deacetylating PAF53, another component of RNA polymerase I complex: PAF53 hypo-acetylation correlates with increased Pol I occupancy on rDNA and transcriptional activation (12).

SIRT7 also regulates the conversion between rDNA transcriptional activation and inhibition during the cell cycle. Here, SIRT7 is phosphorylated by the CDK1–cyclin B pathway during mitosis, rendering SIRT7 inactive such that rDNA transcription is inhibited. Upon exiting mitosis, SIRT7 is dephosphorylated by a phosphatase sensitive to okadaic acid such that rDNA transcription can resume (43).

SIRT7 is also involved in pre-rRNA processing and rRNA maturation. SIRT7 can deacetylate U3-55k, a component of the U3 small nucleolar RNP (snRNP) complex, which increases the association between U3-55k and U3 snRNP. This association is essential for processing pre-rRNA. Under stress conditions however, SIRT7 is released from the nucleoli, resulting in U3-55k hyper-acetylation and reduced pre-rRNA processing (11).

A role for SIRT7 in protein synthesis has also been suggested, as supported by Tsai et al. who found that SIRT7 knockdown suppresses both RNA and protein synthesis (25). They showed that SIRT7 associates with mTOR and TFIIIC2 to modulate Pol III-dependent tRNA transcription, and co-localizes with Pol III target genes. Supporting these observations, SIRT7 knockdown decreased tRNA levels and amino acid incorporation rates; however, SIRT7 over-expression did not increase the rate of protein synthesis, indicating that the observed reduction in protein synthesis may be an indirect effect of SIRT7 knockdown. In addition, the reduced amino acid incorporation rates in SIRT7 knockdown cells were due to reduced abundance of tRNAs for different amino acids (25).

Shin et al. found that Myc depletion significantly reduces SIRT7 occupancy at the promoters of ribosomal proteins. SIRT7 is targeted to the promoters of ribosomal proteins by interacting with Myc and repressing Myc-dependent expression (38).

Finally, SIRT7 may have a role in Pol II activation, as data indicate that SIRT7 associates with Pol II and regulates transcription of snoRNAs and other Pol II genes (17). Here, SIRT7 promotes the release of the elongation factor P-TEFb from the inactive 7SK snRNP complex and deacetylates CDK9, a component of the P-TEFb complex. CDK9 deacetylation promotes Pol II C-terminal domain (CTD) phosphorylation and transcription elongation (17).

### Regulator of cellular stress

SIRT7 is resistant to various cellular stressors, such as endoplasmic reticulum (ER) stress, oxidative stress, mitochondrial protein folding stress, nutrition stress and genotoxic stress (8, 12, 38, 46). As such, it can be inferred that SIRT7 has an important role in regulating cell survival. The function of SIRT7 in regulating rRNA and protein synthesis also supports a role for SIRT7 in cellular stress, because both processes are reduced under stressed conditions.

#### ER stress

The ER is an important intracellular organelle where protein synthesis, folding, modification and trafficking occur. The accumulation of unfolded proteins or depletion of calcium stores triggers the ER stress response (also known as the unfolded protein response, UPR), to restore protein homeostasis by increasing the expression of molecular chaperones, decreasing protein translation and degrading unfolded proteins (47).

SIRT7 can relieve ER stress in two ways. First, under stress conditions, XBP1 induces SIRT7 expression, which in turn reduces ER stress response protein expression, such as CHOP, XBP1s, and GRP78 (38). Second, SIRT7 can interact with Myc to facilitate its recruitment to the promoters of ribosomal proteins, such as RPS20 and RPS14 to repress their gene expression (38).

#### Mitochondrial stress

The mitochondrion is an important organelle for regulating cellular energy homeostasis, and is thus sensitive to many stresses. Cellular stress leads to an accumulation of unfolded mitochondrial proteins resulting in mitochondrial protein folding stress (PFS^mt^) and the unfolded protein response in mitochondria (UPR^mt^) (48). Mohrin et al. found that SIRT7 alleviates PFS^mt^ by repressing NRF1 activity and reducing the expression of the mitochondrial translation machinery (46). NRF1 is a master regulator of mitochondria, and by interacting with SIRT7, targets it to the promoters of mitochondrial ribosomal proteins (mRPs) and mitochondrial translation factors (mTFs) to repress their expression. This mechanism helps alleviate PFS^mt^ and improve cellular survival under conditions of nutrient deprivation (46).

#### Oxidative stress

Many diseases and disorders have been linked with a cellular oxidant–antioxidant imbalance. Data suggest that sirtuins are important in the homeostasis of cellular oxidation–reduction systems. Hypoxia-inducible factors HIF-1 and HIF-2 are essential transcription factors that mediate adaptation to hypoxia (49). Hubbi et al. found that SIRT7 interacts with HIF-1α and HIF-2α proteins and negatively regulates their expression (50). Over-expression of SIRT7 reduced the levels of HIF proteins as well as their transcriptional targets, independent of SIRT7 deacetylase activity and hydroxylation-mediated ubiquitinylation and the proteasomal and lysosomal-mediated degradation pathways. Vakhrusheva et al. found that SIRT7-deficient primary cardiomyocytes exhibit a drastic increase in basal apoptosis compared to wild-type primary cardiomyocytes upon exposure to hydrogen peroxide, suggesting a critical role for SIRT7 in regulating the oxidative stress response and cell death in the heart (8). The researchers speculated that this susceptibility of SIRT7 mutant cells to apoptosis may be due to hyperactive p53, as SIRT7 deacetylates p53 (8). Lewinska et al. also reported that vascular smooth muscle cells exposed to curcumin to induce oxidative damage, exhibited down-regulated SIRT7 and p53 stability (51). SIRT7 down-regulation also decreased Pol I mediated transcription, and the stabilized p53 activated its target protein p21, resulting in cell-cycle arrest. Thus, SIRT7 has a potential role in the resistance to different conditions of oxidative stress.

#### Cardiac injury

SIRT7 has a role in cardiac homeostasis as illustrated by SIRT7 knockout mice that suffer from degenerative heart hypertrophy, as evidenced by cardiac cell fibrosis and inflammation, resulting in inflammatory cardiomyopathy (8). SIRT7 knockout mice also show increased blood lactate levels and decreased endurance to physical activity, due to oxygen insufficiency and decreased oxygen consumption by cardiac muscles stemming from mitochondrial respiratory dysfunction (13). A possible pathway by which SIRT7 maintains cardiac health may be through GABP. SIRT7 promotes GABP complex formation and activation by deacetylating GABPβ1 to enhance the expression of mitochondrial genes and promote mitochondrial respiration (13).

Araki et al. described another SIRT7 pathway that may regulate cardiac health (52). The researchers noted that SIRT7 expression increases at active wound healing sites upon acute cardiovascular injury and thus speculated that SIRT7 may be involved in tissue repair. Consistently, SIRT7 depletion led to reduced collagen production and insufficient angiogenic and inflammatory responses, resulting in impaired wound healing (52). TGF-β is essential to wound healing as it regulates fibroblast chemotaxis, differentiation and the epithelial-to-mesenchymal transition (EMT). TGF-β receptor protein 1 (TβR1) is an important component of the TGF-β signaling pathway (53). Researchers showed that SIRT7 depletion decreases TβR1 levels and reduces downstream signaling. The effect on TβR1, however, was indirect as SIRT7 did not interact with TβR1, but the mediator PICK1 (protein interacting with protein kinase C, alpha), which interacts with TβR1 and SIRT7 together. This study also showed that loss of SIRT7 activates autophagy and PICK1, again affecting TβRI status. Thus, the researchers concluded that SIRT7 maintains TβRI protein levels by modulating autophagy and PICK1 to regulate the TGF-β signaling pathway. In this way, SIRT7 can participate in scar formation, angiogenesis, inflammation and wound healing in response to acute cardiovascular injury (52). Based on these data, SIRT7 may be considered as a good predictor or therapeutic target for cardiac diseases.

### Genome stability

**Figure 3:**
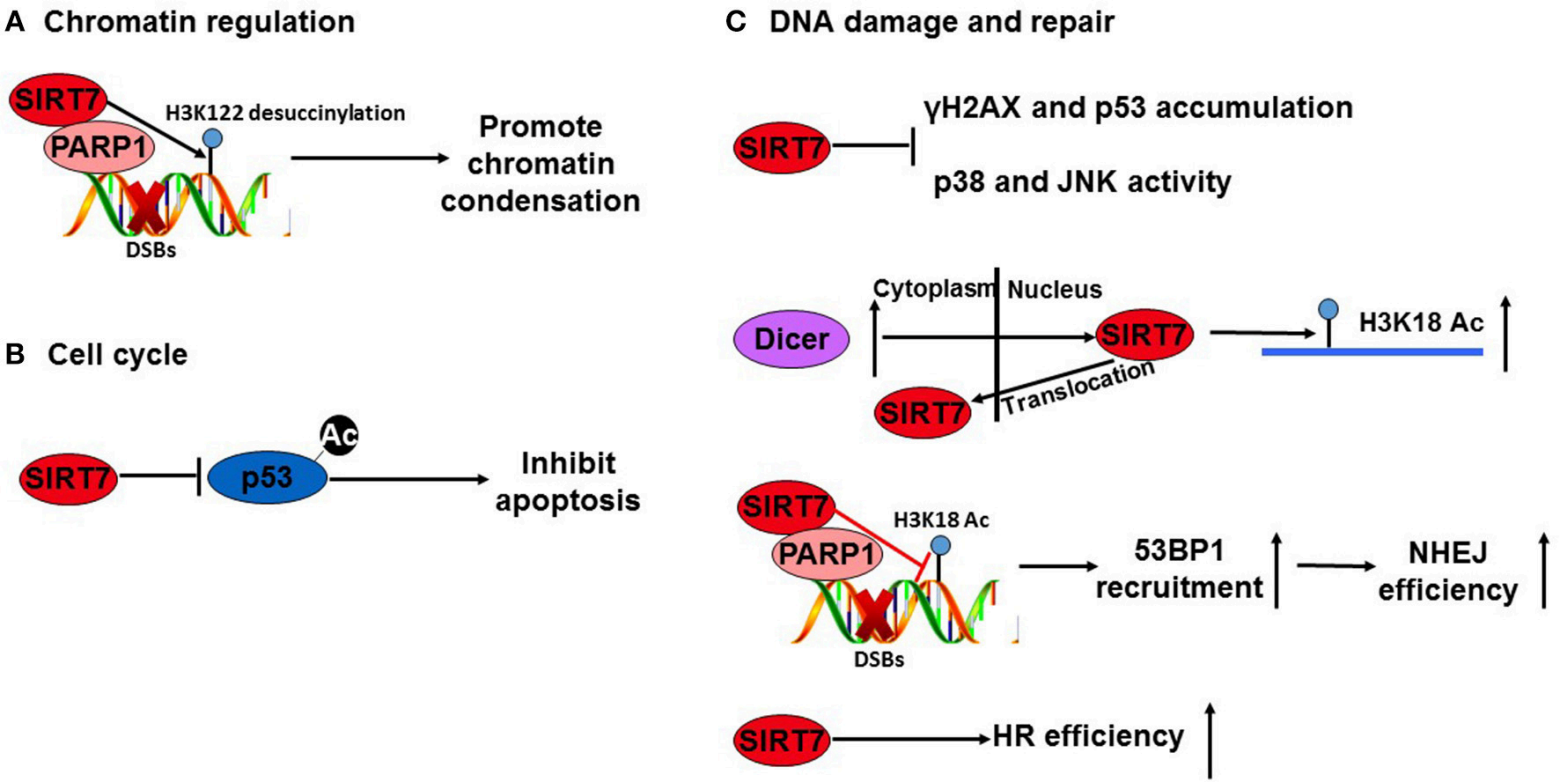
SIRT7 maintains genome stability through multiple pathways. Under stress conditions, SIRT7 participates in the maintenance of genome stability via multiple mechanisms. **(A)** SIRT7 is recruited to DSBs in a PARP1-dependent manner to catalyze desuccinylation of H3K122 at DSBs to promote chromatin condensation. **(B)** SIRT7 can deacetylate p53 to inhibit apoptosis. **(C)** SIRT7 exerts a protective role during genomic insults by attenuating the DNA damage response (preventing γH2AX and p53 accumulation) and the stress-activated MAPK pathway (p38 and JNK activity inhibition). The upregulation of Dicer in cytoplasm promotes SIRT7 translocation from the nucleus to the cytoplasm, thereby causing a decrease in chromatin-associated SIRT7 and elevated H3K18Ac levels. Besides, SIRT7 overexpression increases the efficiency of non-homologous end joining (NHEJ) and homologous recombination (HR). The recruitment of SIRT7 to sites of DNA damage by a PARP1-dependent manner also modulates H3K18Ac levels, thereby influencing 53BP1 recruitment to DNA double-strand breaks (DSBs) to increase the efficiency of NHEJ.

Sirtuins, including SIRT7, maintain genomic stability under stress conditions through a variety of mechanisms (24, 31, 54, 55). SIRT7 protects the genome largely by influencing chromatin structure, cell-cycle progression and DNA damage signaling and repair (Figure 3). SIRT7 knockout mice show an aging-like phenotype, associated with an increased sensitivity to oxidative and genotoxic stress, suggesting a link between SIRT7 and genomic protection (8).

SIRT7 can maintain genomic stability by regulating p53 or DNA damage repair. With regards to p53, Vakhrusheva et al. found that SIRT7 knockout mouse embryonic fibroblasts (MEFs) undergo higher levels of apoptosis than control MEFs upon adriamycin treatment. This process is as a result of p53 hyperacetylation in the absence of SIRT7 (8). Kiran et al. found that SIRT7 exerts a protective role during genomic insults by attenuating the DNA damage response (preventing γH2AX and p53 accumulation) and the stress-activated MAPK pathway (p38 and JNK activity inhibition) (31). Moreover, they found that SIRT7 transfers from the nucleolus to the nuclear matrix after doxorubicin chemotherapy (31). Similarly, Zhang et al. found that SIRT7 translocates from the nucleus to the cytoplasm in response to DNA damage. This translocation occurs through an interaction with Dicer, causing a decrease in chromatin-associated SIRT7 and elevated H3K18Ac levels (56).

Regarding DNA damage repair, Mao et al. found that SIRT7 overexpression increases the efficiency of non-homologous end joining (NHEJ) by 1.5-fold and homologous recombination (HR) by 2.8-fold in paraquat toxin-treated human fibroblast cells (57). A more recent study by Vazquez et al. elucidated a mechanism by which SIRT7 contributes to NHEJ. They demonstrated that SIRT7 is recruited to sites of DNA damage in a PARP1-dependent manner, where it modulates H3K18Ac levels, thereby influencing 53BP1 recruitment to DNA double-strand breaks (DSBs) to increase the efficiency of NHEJ (55). These data provide direct evidence for a role of SIRT7-mediated H3K18 deacetylation in maintaining genome integrity by DSB repair. At the same time, Li et al. also found that SIRT7 is recruited to DSBs in a PARP1-dependent manner, but showed that it catalyzes desuccinylation of H3K122 at DSBs to promote chromatin condensation and efficient DSB repair (24). These latest findings extend our understanding as to how SIRT7 helps maintain genome stability.

### Metabolic regulation

#### Glucose metabolism

The enzymatic activity of SIRT7 depends on its metabolic co-substrate NAD^+^, thus connecting its role to cellular metabolic status. SIRT7 is a low glucose stress sensor that, as discussed, modulates rDNA transcription to preserve energy and resist nutritional stress. Chen et al. identified that SIRT7 redistributes from the nucleoli to the nucleoplasm upon glucose deprivation or treatment with AICAR (a low energy mimic) (12). At the mechanistic level, PAF53 interacts with Pol I and recruits it to rDNA promoters (58). SIRT7 deacetylates PAF53, which acts as a signal to recruit RNA polymerase I to rDNA promoters and activate RNA Pol I-mediated transcription. Under low glucose conditions, the redistribution of SIRT7 from the nucleoli to the nucleoplasm permits PAF53 hyper-acetylation. This hyper-acetylation impairs the interaction between PAF53 and Pol I and decreases Pol I activity, thus leading to rDNA transcription inhibition (12). Sun et al. also showed that glucose starvation induces SIRT7 redistribution via AMPK-directed SIRT7 phosphorylation. This effect causes REGγ-proteasome-dependent degradation, thereby reducing rDNA transcription to avoid cell death (44).

Recent studies have provided insight on the role of SIRT7 in glycolysis. SIRT7 deacylates phosphoglycerate kinase 1 (PGK1), a key enzyme in glycolysis pathway, and suppresses PGK1 enzymatic activity in liver cancer cells (15). We also reported that SIRT7 regulates gluconeogenesis by modulating G6PC expression via USP7-mediated deubiquitination (45). SIRT7 also suppresses HIF1 and HIF2, which repress glucose oxidation through the tricarboxylic acid cycle (59). Taken together, these findings implicate SIRT7 in glucose metabolism.

#### Lipid metabolism

The evidence supporting a role for SIRT7 in lipid metabolism in the liver is conflicting (60). Yoshizawa et al. found that SIRT7 knockout mice (generated by deleting exons 4–9), are resistant to high-fat diet (HFD)-induced fatty liver, glucose intolerance and obesity (61). They also showed that liver-specific SIRT7 knockout mice have reduced hepatic triglyceride accumulation (61). SIRT7 activates Cd36 expression, which is vital for fatty-acid uptake, as well as Mogat that incorporates fatty acids into triglycerides, and Cidea and Cidec that are involved in lipid storage and lipid droplet formation. The expression levels of these four genes were all reduced in the livers of the SIRT7 knockout mice fed a HFD (61). Mechanistically, an interaction between the E3 ubiquitin ligase complex (DDB1-CUL4-associated factor 1 (DCAF1)/damage-specific DNA binding protein 1 (DDB1)/cullin 4B (CUL4B) complex) and TR4 (a nuclear receptor involved in lipid metabolism) promotes TR4 degradation. However, SIRT7 binding to the DCAF1/DDB1/CUL4B complex inhibits TR4 degradation and activates TR4 target genes to increase fatty-acid uptake and triglyceride synthesis and storage. Consequently, the expression level of TR4 and its target genes are reduced in liver-specific SIRT7 knockout mice and lipid synthesis and storage is decreased to resist hepatic steatosis (61).

Two other groups have reported opposing results to Yoshizawa et al. finding that SIRT7 knockout mice instead suffer hepatic steatosis (13, 38). Shin et al. found that SIRT7 knockout mice (generated by replacing exons 4–11 with a LacZ gene), have a fatty liver without obesity (38). They showed that loss of SIRT7 increases lipogenic gene expression, liver triglyceride levels and inflammatory markers, indicating progression to steatohepatitis. These mice also exhibited low levels of plasma triglycerides compared to wild-type controls, due to reduced very-low-density lipoprotein (VLDL) secretion. Importantly, liver steatosis was reversed in these animals by reintroducing SIRT7 specifically in the liver (38). At the mechanistic level, it seems that SIRT7 prevents the development of fatty liver disease by suppressing ER stress. Consequently, SIRT7 knockout mice fail to relieve ER stress such that the UPR pathway is activated causing apoptosis, inflammation, increased lipogenesis and reduced VLDL secretion specifically in the liver (38).

Ryu et al. also described that SIRT7 knockout mice (generated by deleting exons 6–9) exhibited hepatic microvesicular steatosis, and increased plasma levels of triglycerides and free fatty acids. These mice also showed signs of multi-systemic mitochondrial dysfunction due to GABPβ1 hyperacetylation in the absence of SIRT7, including increased blood lactate levels and reduced exercise performance. As discussed, SIRT7 mediates mitochondrial function by deacetylating GABPβ1 (a regulator of multiple nuclear-encoded mitochondrial genes) to promote the formation and activation of the GABP complex that induces mitochondrial gene expression and contributes to mitochondrial homeostasis (13).

Although they used the same mice strain (C57BL/6J), the contradiction of the roles of SIRT7 in lipid metabolism discussed in these three studies may be due to the differences in the genetic background of and construction of SIRT7 knockout mice (60). Further studies with more sample size and parallel experiments are required to clarify this picture.

Cioffi et al. found that SIRT7 induces differentiation and maturation of early adipocyte precursors to promote adipogenesis (39). Consistently, SIRT7 knockdown resulted in reduced Oil Red O staining and adipogenesis marker (FABP4, PPARγ, C/ebpα, adipoq) expression. The researchers also found that miR-93 prevents adipogenesis by inhibiting SIRT7: adipogenesis was enhanced in mir-25-93-106b^−/−^ mice but repressed in miR-93-reintroduced mice. Interestingly, SIRT7 expression was enhanced in the mir-25-93-106b^−/−^ mice while nuclear SIRT7 expression was reduced upon injection of miR-93 mimics into the visceral fat pads of leptin-deficient (ob/ob) mice (39). Despite these preliminary findings, the definitive mechanism underlying how miR-93 regulates SIRT7 and how SIRT7 promotes adipogenesis remains unclear. Fang et al. demonstrated that SIRT7 can promote adipogenesis by binding to SIRT1 and inhibiting its activity by preventing its auto-deacetylation. SIRT1 is reported to repress PPARγ through interaction with nuclear receptor corepressor 1 (NCoR1, PPARγ corepressor) to inhibit adipogenesis (62). As such, SIRT7 knockout mice possess a notably diminished proportion of white fat due to enhanced SIRT1 activity, which blocks PPARγ and adipocyte differentiation (63). SIRT1 activity depletion restores adipogenesis in Sirt7 knockout mice (63). These data implicate a potential cross-regulatory network within the sirtuin family.

#### SIRT7 in mitochondrial metabolism

As discussed, the mitochondrion is a crucial organelle involved in regulating cellular energy homeostasis and thus cell survival. Mitochondria uptake energy from nutrients and then convert it into ATP by oxidative phosphorylation (OXPHOS) (64). In response to cellular stress, the mitochondrion has an armamentarium of quality-control mechanisms based on mitochondrial biosynthesis, mitophagy and mitochondrial unfolded protein responses, to maintain proper mitochondrial function. SIRT7 is an important regulator of mitochondrial homeostasis. As stated above, Ryu et al. found that SIRT7 knockout mice show multi-systemic mitochondrial dysfunction, including lactate accumulation in the blood and age-related hearing loss (13). Mechanistically, SIRT7 impacts on mitochondria function by deacetylating GABPβ1, which forms a hetero-tetramer complex with GABPα, occupies nuclear-encoded mitochondrial target genes and promotes their transcription. Further studies also found that SIRT7 deficiency affects OXPHOS in the heart and liver (13). Mohrin et al. also reported that SIRT7 interacts with NRF1 (a master regulator of mitochondria) to regulate mitochondria size and activity of the mitochondrial respiratory-chain (46).

**Figure 4:**
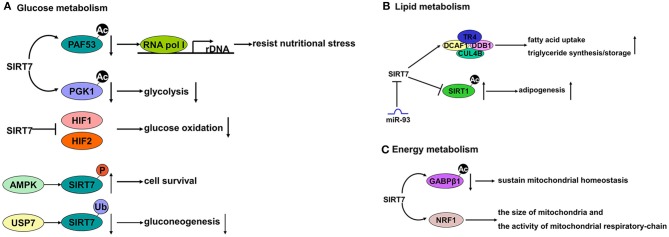
The roles of SIRT7 in metabolism. **(A)** SIRT7 can deacetylate RNA polymerase I (Pol I)-associated factor PAF53 to resist nutritional stress and deacetylate Phosphoglycerate kinase-1 (PGK1) to affect glycolysis pathway. SIRT7 also suppresses the transcription factor Hypoxia-inducible factor-1 (HIF1) and HIF2 to influence glucose oxidation. In addition, SIRT7 can be modified by AMPK or USP7 to regulate glucose metabolism. **(B)** The interaction between SIRT7 and DCAF1/DDB1/CUL4B complex inhibits degradation of Testicular orphan nuclear receptor 4 (TR4) and activates TR4 target genes subsequently increasing fatty acid uptake and triglyceride synthesis/storage. SIRT7 also interacts with SIRT1 to promote adipogenesis. Besides, inhibition of SIRT7 by miR-93 represses adipogenesis. **(C)** SIRT7 can impact the mitochondria function by deacetylating GA-binding protein β1 (GABPβ1). Besides, the interaction of SIRT7 with Nuclear respiratory factor 1 (NRF1) regulates the size of mitochondria and the activity of mitochondrial respiratory-chain.

Overall, SIRT7 has a vital role in metabolic homeostasis (Figure 4), but there remain many unresolved questions. The contradictory effect of SIRT7 on lipid metabolism also needs clarification. Future studies need to address these issues and determine whether SIRT7 may be a suitable therapeutic target in metabolic disorders.

### Aging and senescence

Consistent with the known functions of sirtuins in senescence and lifespan-extension, numerous studies have established a close relationship between SIRT7 and age-related processes (36). SIRT7-knockout mice have a shorter lifespan than control mice, and succumb to premature aging phenotypes around 1 year, with symptoms, such as kyphosis, decreased gonadal fat pad content, reduced IGF-1 plasma levels, hepatic steatosis, degenerative heart hypertrophy, reduced hearing and reduced hematopoietic stem cell–regenerative potential (13, 46, 65). SIRT7 expression gradually declines with age in mice, rat and several cells, including endothelial cells, fibroblasts, hepatocytes and HEK293FT cells (13, 14, 66–68). Kiran et al. found that SIRT7 over-expressing cells show a mostly normal morphology, with very few enlarged cells upon treatment with a low dose of doxorubicin (a cellular senescence inducing agent) (69). By contrast, control GFP expressing cells exhibit cell enlargement and multi-nucleation (typical features of cellular senescence) following doxorubicin exposure. The researchers also found that p53 and p21 senescence marker expression decreases in SIRT7 overexpressing cells upon doxorubicin treatment compared to control cells.

Lee et al. reported that the acetylation levels of nucleophosmin (NPM1) are increased while SIRT6 and SIRT7 levels are decreased in senescent cells. SIRT7 can deacetylate NPM1, which results in up-regulated p53 transcriptional activity in MEFs to induce cellular senescence (14, 70).

Telomeres have the potential to serve as the biomarker of biological cell age (71). Conomos et al. found that TR4 recruitment to the telomere can attribute to the ALT (alternative lengthening of telomeres) phenotype (72). SIRT7 positively regulates the protein level of TR4 (19), suggesting that SIRT7 might play an upstream role in DNA repair and telomere maintenance pathways.

A role for SIRT7 in aging and senescence has also been attributed to SIRT7-mediated regulation of mitochondrial ribosomal proteins (mRPs). SIRT7 interacts with NRF1 to repress mRPs, resulting in hematopoietic stem-cell longevity (46). Conversely, SIRT7-mediated GABPβ1 deacetylation promotes the formation and activation of the GABP complex to increase mRPs, resulting in hematopoietic stem-cell aging (13).

One study reported an interaction between SIRT7 with Tripeptidyl peptidase II (TPPII) that permits SIRT7 cytoplasmic localization (52). TPPII has regulatory effects on apoptosis and senescence, as observed in TPPII knockout mice that exhibit early immuno-senescence and have a shorter lifespan than controls (73). SIRT7 may, therefore, regulate aging and senescence by interacting with TPPII, but the underlying mechanisms need further study.

rDNA instability is common in premature aging syndromes. A recent study by Paredes et al. uncovered a critical role for SIRT7 in protecting against cellular senescence by maintaining heterochromatin silencing and rDNA stability. Mechanistically, they found that SIRT7 acts as a scaffold to stabilize SNF2H at rDNA promoters for chromatin silencing (74). Taken together, SIRT7 might prevent aging-induced physiological changes and possibly extend lifespan via numerous pathways.

### Cancer

**Figure 5:**
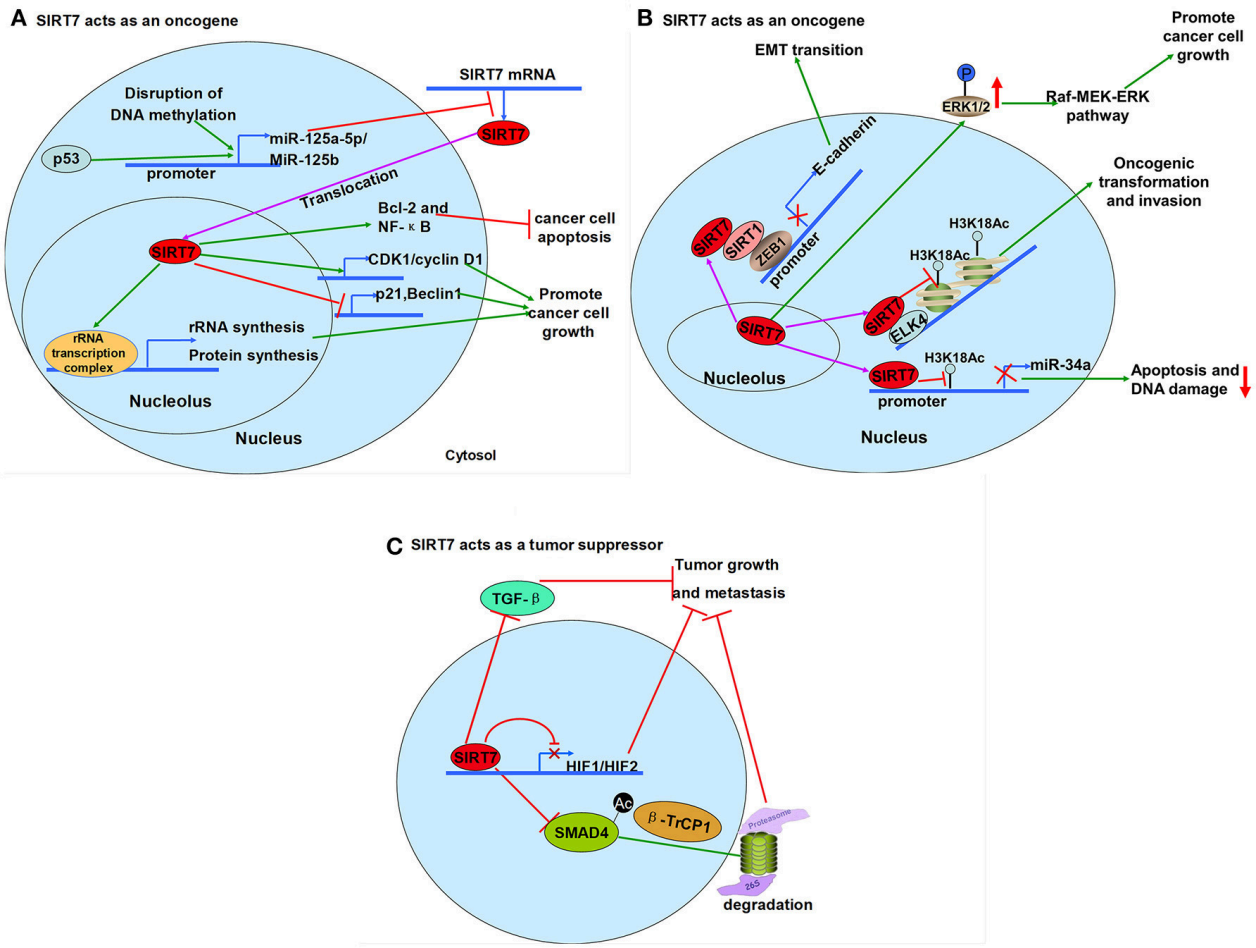
The roles of SIRT7 in cancer. **(A,B)** SIRT7 acts as an oncogene. **(A)** The disruption of DNA methylation and p53 activates miR-125b and miR-125a-5p to inhibit the expression of SIRT7. Overexpression of SIRT7 upregulates the expression of CDK1/cyclin D1 while downregulates the level of p21 and Beclin1 as well as promotes rRNA and protein synthesis by interaction with rRNA transcription complex to promote cancer cell growth. In addition, overexpression of SIRT7 can also upregulate Bcl-2 and NF-κB to repress cancer cell apoptosis. **(B)** ETS-like transcription factor 4 (ELK4) targets SIRT7 to promoters of many tumor suppressor genes for H3K18 deacetylation and leads to cancer cell growth and metastasis eventually. SIRT7 induces ERK1/2 phosphorylation and activated the Raf-MEK-ERK pathway to promote cancer cell growth. SIRT7 also reduces miR-34a expression by deacetylating H3K18Ac to decrease cancer cell apoptosis and DNA damage. Besides, SIRT7 reduces expression of E-cadherin by interaction with SIRT1 to promote EMT transition. **(C)** SIRT7 acts as a tumor suppressor. SIRT7 inactivates TGF-β signaling and represses epithelial-to-mesenchymal transition. SIRT7 also negatively regulates HIF1 and HIF2 transcription. Moreover, SIRT7 deacetylates Mothers against decapentaplegic homolog 4 (SMAD4) and promotes β*-*transducin repeat–containing protein 1 (β-TrCP1)-dependent degradation to inhibit tumor growth and metastasis.

Although ample evidence supports the involvement of SIRT7 in carcinogenesis, SIRT7 exhibits opposing roles in different cancer types (Figure 5). SIRT7 is up- regulated in the majority of cancers, including colorectal, gastric, thyroid, node-positive breast, bladder, ovarian and cervical cancers, hepatocellular and epithelial prostate carcinoma, where it acts as an oncogene (9, 40, 75–82). The exception is seen in pancreatic cancer, where SIRT7 is down-regulated and seems to act as a tumor suppressor (83) (Table 2).

**Table 2:** SIRT7 related cancers.

**Cancer**	**SIRT7 expression**	**Role/Impact**
Colorectal cancer	Overexpression	Oncogene
Colorectal cancer	Downregulated	Induce radio-sensitivity; Enhance therapeutic effects
Gastric cancer	Overexpression	Oncogene
Thyroid cancer	Overexpression	Oncogene
Node-positive breast cancer	Overexpression	Oncogene
Breast cancer	Deficiency	Increase metastasis
Bladder cancer	Overexpression	Oncogene; promote cancer-cell growth
Ovarian cancer	Overexpression	Oncogene
Cervical cancer	Overexpression	Oncogene
Hepatocellular carcinoma	Overexpression	Oncogene; promote cancer-cell growth
Epithelial prostate cacinoma	Overexpression	Oncogene; maintaining cancer phenotypes; increase metastasis
Pancreatic cancer	Downregulated	Might be tumor suppressor
Liver cancer	Knockdown	Promote cancer-cell growth

As an oncogene, SIRT7 inhibition reduces cancer-cell growth, represses colony formation and cancer-cell metastasis and increases cancer-cell apoptosis. High SIRT7 expression is also considered a predictor of poor survival in various cancers (9, 40, 75–82). Many studies have reported mechanisms of action by which SIRT7 promotes cancer-cell growth (Figure 5). Yu et al. found that SIRT7 induces ERK1/2 phosphorylation and activates the Raf–MEK–ERK pathway to promote cancer-cell growth (81). Hypoacetylation of H3K18 is associated with oncogenic transformation, aggressive tumor phenotypes and poor prognosis and maintenance of essential human cancer-cell features, including anchorage-independent growth and escape from contact inhibition (84). As shown in prostate cancer, SIRT7 binds and maintains the deacetylated state of H3K18 at the promoters of many tumor suppressor genes by interacting with the ELK4 transcription factor (85, 86). Thus, SIRT7 has a fundamental role in maintaining cancer phenotypes (6).

Zhang et al. found that SIRT7 knockdown promotes gastric cancer-cell apoptosis (82). Mechanically, SIRT7 prevents cellular apoptosis by down-regulating miR-34a via H3K18ac deacetylation. The miR-34 family is associated with cell-cycle arrest, senescence and apoptosis in cancers, and low miR-34a expression is associated with poor prognosis (82).

Another mechanism as to how SIRT7 promotes cancer-cell growth was identified by Kim et al. (9). They found that SIRT7 knockdown increases the number of liver cancer cells in G1/S phase and delays the cell-cycle transition: p21^WAF1/Cip1^ expression was increased and cyclin D1 (a G1/S cell cycle regulator) was suppressed in SIRT7 knockdown cells. In patients with hepatocellular carcinoma, increased SIRT7 expression was attributed to p53 mutation or the endogenous hypermethylation of the microRNAs miR-125a-5p and miR-125b. Over-expression of SIRT7 led to p21^WAF1/Cip1^ suppression and induction of cyclin D1 expression to promote cancer-cell growth (9). Han et al. also found that SIRT7 expression is regulated by hsa-miR-125b in bladder cancer. They observed that in bladder cancer, hsa-miR-125b and SIRT7 are inversely associated with the oncogenic long non-coding RNA MALAT1. Up-regulated hsa-miR-125b resulted in down-regulated SIRT7 and MALAT1; this effect inhibited bladder cancer cell growth, induced apoptosis, and decreased cell motility (40).

With regards to apoptosis, Wang et al. found that shRNA-mediated SIRT7 silencing reduced anti-apoptotic factor B-cell lymphoma 2 (Bcl-2) and nuclear factor kappa B (NF-κB) levels (80). The NF-κB signaling pathway is important in cellular proliferation, apoptosis and migration in malignant diseases (87). The researchers concluded that SIRT7 inhibits cancer-cell apoptosis by up-regulating Bcl-2 and NF-κB levels (80). The precise regulatory mechanisms remain to be identified.

SIRT7 also influences cancer-cell metastasis. High SIRT7 expression is associated with aggressive cancer phenotypes, metastatic diseases and poor patient prognosis; down-regulating SIRT7 reverses the metastatic properties of epithelial and mesenchymal cancer cells. In epithelial carcinomas, SIRT7 is associated with the EMT, a key process in metastatic progression. Malik et al., Yu et al., Zhang et al. focused on classic EMT regulatory factors and discovered that the expression of E-cadherin (an EMT regulatory factor) showed inverse correlation with SIRT7 *in vivo*. In SIRT7-deficient cells, E-cadherin and DAB2 interacting protein (DAB2IP; a tumor suppressor gene, whose loss promotes EMT and metastasis in prostate cancer) are significantly increased in mRNA level. These findings suggest a role for SIRT7 in cancer prevention and as prognostic factor (78, 81, 82). SIRT7 interacts with SIRT1 to enhance SIRT1-dependent prostate cancer cell metastatic properties and promotes E-cadherin transcriptional repression (88).

In non-epithelial cancers, SIRT7 may impact metastasis regulatory pathways to affect cellular metastatic properties. The expression levels of matrix metalloproteinase MMP16 and vascular endothelial growth factor (VEGF-A) are reduced in SIRT7-deficient HT1080 cells compared to control cells (78). Thus, SIRT7 may be of vital importance for cancer-cell metastasis (78).

As a tumor suppressor, low levels of SIRT7 are associated with aggressive tumor phenotypes and poor patient outcomes. One study found that patients with pancreatic cancer and high levels of nuclear SIRT7 had a longer lifespan (succumbed to disease later) than those with low levels of SIRT7. However, the precise mechanism underlying this association is unclear (83). Similar to the other sirtuins, SIRT7 has also been recognized as a tumor suppressor based on its negative regulation of HIF1 and HIF2 transcription (50, 89), as previously discussed.

The contradictory roles of SIRT7 in cancer may be related to its multiple interactions and functions in various cellular processes. First, SIRT7 deacetylates H3K18Ac, whose depletion is associated with highly malignant cancers and poor patient prognosis. Second, SIRT7 influences ribosome biogenesis to meet the high biosynthetic and metabolic needs of cancer cells. At the same time, although SIRT7 inactivates p53 by deacetylation, up to half of all tumors exhibit mutated p53, which may diminish the oncogenic role of SIRT7 and result in tumor suppressive characteristics (83, 90). Even though the definitive function of SIRT7 is uncertain, many consider SIRT7 as a cancer biomarker or a predictor of prognosis (73, 75, 83, 81). In sum, data suggest that SIRT7 may be a potential novel biomarker for prognosis in pancreatic cancer (83), a circulating marker in head and neck squamous cell carcinoma (73), and a predictive biomarker of pancreatic cancer (PCa) aggressiveness (91).

Our lab also found that down-regulation of SIRT7 after 5-FU exposure induces radio-sensitivity in human colorectal cancer and enhances therapeutic effects (92). Shi et al. found the microRNA-3666 induces SIRT7 inhibition, which in turn inhibits non-small cell lung cancer cell growth (41). Recently, Tang et al. found that SIRT7 deficiency promotes breast cancer cell metastasis, while temporal expression of SIRT7 inhibits metastasis in a polyomavirus middle T antigen breast cancer model. Here, SIRT7 deacetylates SMAD4 and promotes β-TrCP1-dependent degradation. Finally, SIRT7 deficiency activates TGF-β signaling and enhances the EMT (21). Therefore, it can be assumed that SIRT7 is a worthwhile target to explore for cancer therapy.

## Conclusions and future perspectives

The function of SIRT7 was ignored in the initial years of sirtuin-based research due to its localization in the nucleolus. However, major breakthroughs have been made over the past decade in SIRT7 biology. New substrates for SIRT7 deacetylase activity have been identified, which have highlighted new enzymatic activities that are critical for different cellular processes. SIRT7 has strong potential as a therapeutic target in various cancers, due to the identification of its up-regulation and activation in various cancer cells. SIRT7 is also considered a potential novel biomarker for prognosis in several cancers, such as pancreatic cancer. However, due to its complicated and controversial mechanism in the maintenance of cancers, future studies are needed to understand the precise molecular mechanism and downstream pathways of SIRT7 in the specific cancer types. The therapeutic uses of SIRT7 in cancers will rely on the further clinical trials. A recent report suggests a role for SIRT7 in the adaptive immune system and neurogenesis (93). This finding indicates that the roles and function of SIRT7 are still diversifying, and are wider than previously thought. Further studies are now needed to better understand and elucidate the molecular role of SIRT7, identify its substrate partners/cofactors, and delineate the intracellular pathways that regulate their activity in different disease models.

## Author contributions

DW, YL, and KZ wrote the primary manuscript and revised the manuscript. HW and W-GZ conceived and designed the manuscript.

### Conflict of interest statement

The authors declare that the research was conducted in the absence of any commercial or financial relationships that could be construed as a potential conflict of interest.
